# Assessing health outcomes in the aftermath of the great recession: a comparison of Spain and the Netherlands

**DOI:** 10.1186/s12939-020-01203-6

**Published:** 2020-06-05

**Authors:** Kristina Thompson, Annemarie Wagemakers, Johan van Ophem

**Affiliations:** 1 Department of Health Sciences, Vrije Universiteit Amsterdam, Amsterdam, the Netherlands; 2 Health and Society Group, Wageningen University & Research, Wageningen, the Netherlands; 3 Urban Economics Group, Wageningen University & Research, Wageningen, the Netherlands

**Keywords:** Wealth, Health, Financial security, Great Recession, Financial crisis, Europe, Spain, Netherlands

## Abstract

**Background:**

Across time and space, financial security has been shown to impact health outcomes, with the acute loss of financial security being particularly detrimental. We compare financial security’s association with health in Spain and the Netherlands. These countries respectively exemplify low and high levels of financial security, general trends that have been exacerbated by the Great Recession of the 2010s.

**Methods:**

We exploit the Spanish (*n* = 1001) and Dutch (*n* = 1010) editions of the European Social Survey 7, conducted in 2014, and condense relevant financial security- and health-related survey questions into latent variables using factor analyses. Using the component loadings as quasi-weightings, we generate one financial security variable and three health variables (mental, physical and social). Then, we run ordinary least squares regressions interacting financial security and nationality, for each of the three health outcomes.

**Results:**

In unadjusted models, we find that financial security (*p* < 0.01) is positively associated with the three health outcomes, while being Spanish relative to being Dutch (*p* < 0.01) is associated with worse health outcomes. However, the results of the interaction term show that being Spanish relative to being Dutch weakens the relationship between physical health and social health, although not mental health.

**Conclusions:**

We find evidence that financial security’s influence on health outcomes may vary in different contexts. This may be an important aspect of determining the Great Recession’s influence on health outcomes. Our study is a first step in understanding how the relationships between financial security and health may differ in countries with different experiences of the Great Recession.

## Introduction

Time and again, wealth has been shown to be a key determinant of health [1, 2]. The greater amount of money individuals have, the healthier they tend to be. Just as a greater amount of wealth has been found to be associated with better health, a lack and/or loss of wealth have been shown to be associated with worse health [3, 4]. A sizeable body of literature is dedicated to this topic (e.g. Cutler, Miller & Norton [5], examining the Great Depression; and Tapia Granados [6] examining post-war Japan). More recently, a number of studies have explored how financial insecurity is associated with health outcomes in Europe in the context of the Great Recession of the 2010s [7, 8].

Likewise, we are interested in exploring financial security’s relationship to health outcomes in Europe in the aftermath of the Great Recession. This context is particularly informative, given the widespread reach of the Great Recession across countries and, often, socio-economic strata [9]. The Great Recession – widely considered to be the most acute financial crisis since the Great Depression – began as a subprime mortgage crisis in the U.S. in 2007, and evolved into a global financial downturn. In the Eurozone, the Great Recession took the form of a sovereign debt crisis, reaching its apex in 2010 and 2011. A number of Eurozone economies - Portugal, Ireland, Italy, Greece and Spain – defaulted on debts, and received bail-outs and/or credit agreements. Throughout Europe, austerity measures were implemented, in part to comply with these agreements. In many instances, austerity compounded the corrosive effects of the Great Recession on households: across the Eurozone, unemployment rates spiked, while consumer spending and household savings rates declined [9]. While many less-affected countries have returned to growth, others continue to be impacted by the Great Recession, particularly on a household level [9]. This has left Eurozone countries in particular with vastly different levels of financial security.

Already, a number of studies examine the relationship of financial security to health outcomes in the wake of the Great Recession, with two systematic literature reviews on the topic [10, 11]. However, both reviews concluded that the extent to which health outcomes are associated with the Great Recession is not yet known. Parmar, Stavropoulou & Ioannidis [10] argued that this may be because of the variable quality of included studies. Thompson, van Ophem & Wagemakers [11] identified potential  sources of this variability: one was that a majority of studies used simple or straightforward measures of wealth and health, possibly failing to accurately and/or completely measure these abstract concepts [11]. Operationalizing multi-faceted definitions of wealth and health would likely overcome this. Another possible issue was the populations in the included studies: nearly all studies in both reviews either examined single countries within Europe, or looked at Europe/the Eurozone as a single bloc, potentially obscuring important region- or country-level differences [11]. One way of understanding if the relationship of financial security and health differed based on country may be to compare a country that fared relatively well in the Great Recession with one that fared relatively poorly. To address both of these shortcomings, we pose the research questions: to what extent is financial security associated with different health outcomes in the aftermath of the Great Recession? And to what extent does financial security’s association with health outcomes vary by country?

### Setting

We select Spain and the Netherlands as informative cases to answer our research questions. Providing some common ground for comparison, both Spain and the Netherlands are Eurozone countries and so have been subject to similar macro-economic policies. Still, these countries differ in ways that profoundly impact their levels of financial security, particularly regarding their social security systems. The Netherlands has among the most comprehensive social security systems in Europe [12, 13]. Spain, on the other hand, has a well-developed single payer healthcare system, but a social security system that is comparatively limited regarding unemployed benefits [14, 15].

Moreover, the Great Recession was much more acute in Spain than in the Netherlands. Dutch real gross domestic product (GDP) growth was negative in 2009, 2012 and 2013. But by 2015, growth had surpassed pre-crisis (2008) levels [16]. In contrast, Spain experienced 5 years of negative or flat growth, although growth figures in the years since 2014 have approached pre-recession figures [16].

These differences in national-level wealth were borne out on the household level. For instance, unemployment was (and continues to be) significantly less of a widespread problem in the Netherlands than in Spain. The Netherlands’ unemployment rate rose as a result of the Great Recession, but to a relatively modest 7.4% in 2014 [17]. This compares to 11.6% in the Eurozone in the same year [17]. In contrast, Spain’s unemployment rate was among the highest in Europe, climbing to 26.1% in 2013 and 24.5% in 2014. Spain’s unemployment problem was particularly acute for young people: the youth unemployment rate was above 50% in 2013 and 2014 [17]. The Netherlands’ youth unemployment rate fluctuated between 12 and 13% over the same period. More generally, most measures of individual or household wealth indicate that the Netherlands has fared better than most other Eurozone countries, while Spain has fared worse [18]. Table 1 includes various measures of macro- and household- level financial well-being in Spain, the Netherlands, and for sake of comparison, the Eurozone/Europe overall. Broadly speaking, not only is there less support for the unemployed in Spain than the Netherlands, there are also far more people who are unemployed and/or in precarious financial positions in Spain than in the Netherlands.

**Table 1:** Financial security indicators

Indicator	Spain	Netherlands	Eurozone
GDP growth, 2014 [16]	1.4%	2.0%	2.0%
Unemployment rate, 2014 [17]	24.5%	7.4%	11.6%
% at risk of poverty, 2014 [18]	28.6%	16.4%	23.4% (includes EU-28)
Household savings rate, 2015 [18]	9.0%	14.5%	10.7%
Gini coefficient, 2013 [19]	0.346	0.283	0.300

### Theoretical framework

A great deal of literature has grappled with the abstract concepts of financial security and health. First looking at financial security, we argue that the amount of money people feel they need is just as important (if not more important) than the amount of money they actually have [20]. We define financial security as a composite of income security (how much money people actually have, and how people feel their income enables them to participate in society), job security (how likely people feel that they will be able to keep their jobs) and housing security (how likely people feel they will be able to keep their homes) [21, 22]. These measures encompass the material and non-material aspects of financial well-being.

Next, looking at health, we employ Huber et al. [23]‘s definition, which states that health is “the ability to adapt and self-manage in the face of social, physical, and emotional challenges.” Here, coping with change is the focus, and includes elements of mental, physical and social well-being. Huber et al.’s definition also views health as a spectrum, without a binary between health and disease [23]. We therefore define health overall as being composed of mental, physical and social aspects.

It is also worth noting that different aspects of health have been shown to be differently affected by changes in financial security. For instance, measures of mental and self-rated health have been shown to rapidly deteriorate in response to financial crises [24, 25]. In contrast, measures of physical health (particularly mortality rates) seem to improve in the wake of recessions [26]. Further, in some cases, social health has been shown to be a protective factor against worsening mental and physical health during recessions [27].

With these understandings of financial security and health, we hypothesize that the financial security is negatively associated with social, physical and mental health. We also hypothesize, based on the various measures of financial security outlined in the “Setting” section, that the relationship between financial security and health is moderated by nationality, and is stronger in Spain than in the Netherlands.

## Methods

We exploit the European Social Survey (ESS), and condense relevant questions into the latent constructs of financial security and health via factor analyses, resulting in one financial security and three health (mental, physical, social) variables [28]. We then run ordinary least squares (OLS) regressions, with each of the health variables as an outcome. For each of the three health outcomes, Models 1 and 2 look at the unadjusted associations of financial security and nationality (being Spanish relative to being Dutch), respectively, on the health outcomes. Models 3 (without additional covariates) and 4 (with additional covariates) explore the interactions of financial security and nationality on the health outcomes.

### Data

#### Sample

The present study exploits the routine dataset, the ESS. It is a publicly available, nationally representative, survey-based cross-sectional dataset of European countries that has been conducted every 2 years since 2002. One of the most recent editions, the ESS7 was conducted in 2014 and contains data from 21 countries [28]. While all editions of the ESS contain information on social conditions in Europe, the ESS7 is particularly useful for our purposes because it includes a rotating module on health inequalities. There are 1925 research persons in the Spanish dataset, and 1778 in the Dutch dataset. However, because the ESS contains a sample of research persons 15 years and older, the sample contains cases who are not of typical working-age. We opt to only include research persons who are (roughly) working-age, so that all research persons aged 24 and under and 66 and older are excluded. Also, because we are interested in the influence of being Spanish or Dutch on health outcomes, we only include nationals of these countries. This results in a sample of 1001 research persons in Spain and 1010 research persons in the Netherlands.

#### Variables

##### Outcome and key predictor variables

The key predictor (financial security) and outcome (health) variables are generated via factor analyses. To do so, questions thought to best reflect the concepts of financial security, mental, physical, and social health are selected. Table 2 contains the full list of questions, including the financial security and health components that they are expected to form, their original phrasing in the ESS, and their question numbers. All variables are rescaled so that a lower score is associated with a worse interpretation, while a higher score is associated with a more positive one. For instance, question C7 (‘How is your health in general?’) was originally coded so that 1 = very good health, and 5 = very bad health. We have recoded this so that 1 = very bad health, and 5 = very good health. For ease of interpretation, we also rescale continuous and ordinal variables so that they do not contain zero. Finally, we use a single binary indicator for research persons having one or more of the four illnesses asked in question E30. This is because these illnesses have similarly been linked to environmental and lifestyle factors. Further, these illnesses are in many cases comorbid: of the research persons who stated that they have one of these illnesses, 21% indicated two or more. Table 2 reflects these changes.

**Table 2:** Underlying questions of key predictor and outcome variables

Expected component	Variable name	Scale	ESS question
Financial security	Using this card, please tell me which letter describes your household’s total income, after tax and compulsory deductions, from all sources?	1 = first decile; 10 = tenth decile	F41
	Which of the descriptions on this card comes closest to how you feel about your household’s income nowadays?	1 = very difficult on present income; 4 = very easy on present income	F42
	What was your main activity in the past 7 days?	1 = employed; 0 = unemployed	F17a
Mental health	Taking all things together, how happy would you say you are?	1 = very unhappy; 10 = very happy	C1
	How satisfied are you with your life as a whole?	1 = very unsatisfied; 10 = very satisfied	B20
	When was the last time you felt depressed?	1 = all or almost all of the time; 4 = none or almost none of the time	E20
	When was the last time you felt that everything you did was an effort?	1 = all or almost all of the time; 4 = none or almost none of the time	E21
	When was the last time your sleep was restless?	1 = all or almost all of the time; 4 = none or almost none of the time	E22
Physical health	How is your health in general?	1 = very bad; 5 = very good	C7
	Are you hampered in your daily activities in any way by any longstanding illness, or disability, infirmity or mental health problem?	1 = yes, a lot; 2 = yes, somewhat; 3 = no	C8
	Have you or ever have you had the health problems listed on the showcard? (diabetes, heart/circulation problems, breathing problems or high blood pressure)	0 = yes; 1 = no	E30
Social health	Compared to other people of your age, how often would you say you take part in social activities?	1 = much less than most; 5 = much more than most	C2
	How often do you meet socially with friends, relatives or work colleagues?	1 = never; 7 = every day	C4
	How many people, if any, are there with whom you can discuss intimate and personal matters?	1 = none; 6 = 10 or more	C3

##### Additional covariates

To answer our second research question, we explore the influence of being Spanish (=1) or Dutch (=0) on health outcomes. We anticipate that being Spanish relative to being Dutch is negatively associated with health outcomes, and test this unadjusted relationship in Model 2 [29, 30]. We also anticipate that financial security varies based on citizenship, so we include an interaction term of citizenship and financial security in Models 3 and 4.

Further, additional covariates that may confound the relationship between financial security and health are selected. Table 3 presents these variables. In line with the outcome and key predictor variables, these are rescaled. We base our expectations for additional covariates on existing research, and test for confounding using a stepwise inclusion process. These are included in Model 4 for all three health outcomes.

**Table 3:** Included covariates

Variable	Scale	ESS question
Nationality	1 = Spanish; 0 = Dutch	n/a
Gender	1 = man; 0 = woman	F41
Age	Dummies by 10 year birth cohorts	F42
Education	Dummies by education level completed	F15
Living with a spouse or partner	0 = yes; 1 = no	F36
Using the same card, please tell me how often you and your family experienced severe financial difficulties when you were growing up?	1 = Always; 5 = Never	E32

First, we include gender as a confounder. Gender likely impacts financial security, as men in Europe continue to out-earn women by 14.8%.in the Netherlands and 14% in Spain as of 2018 [31]. Gender also likely impacts health. The conventional wisdom is that, while women tend to live longer than men, they are more likely to be chronically ill [32]. Women are also  more likely in Europe to be diagnosed with mental illnesses such as anxiety and depression [33]. Still, women tend to have wider social networks than men. We therefore anticipate that gender will have a negative effect on mental and physical health, but not social health.

We also anticipate that age confounds financial security’s influence on health. On the one hand, older individuals likely are more financially secure, as they have had more time to earn. On the other hand, being older may have a negative impact on health outcomes. Particularly for physical health, older people have been found to have much higher rates of mild to moderate disabilities [34]. Also, older adults tend to socialize less frequently and have narrower social networks than younger adults [35].

Further, we expect education to confound the relationship between financial security and health. We expect that those with higher educational attainments to be more financially secure. Education’s relationship to health outcomes is less straightforward: while education may in part influence health through financial security, it also has been found to influence health outcomes directly [36]. We therefore treat it as a confounder.

We also expect that cohabitating with a spouse or partner confounds the relationship between financial security and health. Married couples, by virtue of having two incomes, tend to be more financially secure. Also, the widely-held view that cohabitating with a spouse or partner is associated with better health outcomes is supported by research [37]. However, being unhappily coupled may produce the opposite effect [38]. We therefore expect living with a spouse to be associated with better health outcomes, but that this effect may be relatively small.

Finally, financial security in childhood has been shown to be a strong predictor of financial security in adulthood [39]. Also, a sizeable body of literature has shown that poverty during development has been linked to poorer health outcomes in adulthood [40]. We therefore expect strong, positive relationships between financial security and the health outcomes.

We also initially tested for variables concerning health behaviors for confounding. We anticipated that lower BMI, greater frequency of sports and vegetable consumption, and lower frequency of binge drinking are associated with better health outcomes, outcomes supported by existing literature [41]. However, these variables do not confound the relationships between financial security and any of the three health outcomes, and so are not included in our analyses.

### Analyses

#### Factor analyses

We use the data analysis package STATA version 16 to analyze the combined Dutch and Spanish datasets of the ESS7. First, KMO and Bartlett’s tests are run to ensure that the data is appropriate for factor analysis. We use factor analyses with polychoric correlation matrices. These have been found to be more suitable for Likert scale-style ordinal data than other (usually Pearson) correlation matrices [42]. Because we assume that the underlying financial security variables and health variables (included in Table 2) are uncorrelated with one another, we run two factor analyses, using oblique rotation. Table 4 presents the component matrix of the financial security factor analysis, while Table 5 presents the component matrix of the health factor analysis.

**Table 4:** Rotated component matrix, financial security

Household income, deciles	0.6235
Feelings about current household income	0.4620
Employment in last 7 days	0.6405

**Table 5:** Rotated component matrix (oblique rotation), health outcomes

	1 Mental health	2 Physical health	3 Social health
How happy are you?	0.7626		
Satisfied with life as a whole	0.7630		
Felt depressed last week, how often	0.6255		
Sleep was restless last week, how often	0.6736		
Couldn’t get going last week, how often	0.6996		
Self-rated health		0.7525	
Hampered in daily activities		0.7212	
Health problems		0.5647	
Meet with friends/relatives/colleagues, how often			0.5259
Take part in social activities relative to peers			0.4364
Number of people to discuss personal matters			0.4778
Variance explained	0.6071	0.5077	0.5033

To generate the financial security and health variables we use in our models, we multiply each variable's loading by the original value of the variable itself, thereby creating a quasi- weighting [43]. Then, we add the resulting weighted variables together, which gives us one variable per component. For the sake of interpretability, we standardize each of these combined variables by converting them to z-scores, so that they each have a mean of 0 and a standard deviation of 1 [43].

#### Regression analyses

To understand the extent to which financial insecurity impacts health, we use OLS regressions. First, we test the unadjusted relationships between financial security (Model 1) and nationality (Model 2) and each of the three health outcomes. Given that we expect nationality to differently impact the relationships between financial security and health, we interact these two variables in Models 3 and 4. While Model 3 only includes financial security, nationality and the interaction of the two, Model 4 includes the confounders identified in the "Additional covariates" sub-section. Model 4 is specified:

$$ \hat{\mathrm{y}}={\upbeta}_0+{\upbeta}_1{\mathrm{FinancialSecurity}}_1+{\upbeta}_2{\mathrm{Nationality}}_2+{\upbeta}_3\mathrm{FinancialSecurity}\ast {\mathrm{Nationality}}_3+{\upbeta}_4{\mathrm{Gender}}_4+{\upbeta}_5{\mathrm{Age}}_5+{\upbeta}_6{\mathrm{Education}}_6+{\upbeta}_7{\mathrm{FinancialSecurity}\mathrm{Childhood}}_7+{\upvarepsilon}_{\mathrm{i}}, $$

where ŷ represents the predicted value of one of the health outcomes, β_0_ represents the constant term, β_1–7_ represent the variables described in the "Outcome and key predictor variables" and “Additional covariates” sub-sections, and ε_i_ represents the error term.

Given that there are three outcome variables and four types of models, we run a total of 12 regressions. We report beta coefficients, whereby a positive coefficient is associated with better health outcomes.

## Results

Descriptive statistics are presented in Table 6. In terms of the outcome health variables, all appear to be negatively skewed. This is also the case for the financial security variable, but less so.

**Table 6:** Descriptive statistics

Variable	Obs.	Mean	SD	Min.	Max.
Outcomes					
Mental health (factor analysis)	2011	0	1	−5.026	1.687
Physical health (factor analysis)	2011	0	1	−4.137	1.221
Social health (factor analysis)	2011	0	1	−3.626	2.643
Key predictors					
Financial security (factor analysis)	2011	0	1	−2.314	1.808
Nationality^a^					
Spain	1001	−0.083	1.062	− 2.314	1.808
Netherlands	1010	0.082	0.928	−2.314	1.808
Additional covariates					
Financial security in childhood (1 = always difficult; 5 = never difficult)	2011	4.087	1.086	1	5
Gender^a^					
Woman	992	−0.029	0.999	−2.314	1.808
Man	1019	0.028	1.000	−2.314	1.808
Age^a^					
25–35 years	465	0.001	0.968	−2.314	1.808
36–45 years	483	−0.001	0.988	−2.314	1.533
46–55 years	540	0.0134	1.065	−2.314	1.808
56–65 years	523	−0.014	0.972	−2.314	1.605
Education^a^					
Lower secondary and below	703	−0.419	1.019	−2.314	1.808
Upper secondary	460	−0.048	0.979	−2.314	1.808
Lower tertiary	451	0.221	0.877	−2.314	1.605
Upper tertiary and above	397	0.546	0.761	−1.836	1.533
Cohabitating with spouse or partner^a^					
Yes	1412	0.168	0.922	−2.314	1.808
No	599	−0.398	1.062	−2.314	1.808

^a^Mean, standard deviation, minimum and maximum based on financial security score

The sample is roughly evenly divided among Spanish (*n* = 1001) and Dutch (*n* = 1010). Research persons from Spain have a lower than average (− 0.083) financial security z-score, while those from the Netherlands have a higher than average (0.082) z-score.

In terms of additional covariates, it appears that a majority of research persons experienced a high level of financial security in childhood, with a mean score of 4.087. Regarding gender, our sample is roughly evenly divided among women (*n* = 992) and men (*n* = 1019). Women have a slightly below-average financial security score (− 0.029), while men’s is slightly above-average (0.028).

In terms of age, all four cohorts have roughly similar financial security scores. In terms of education, the largest share (35%) have a lower secondary education or below. This is followed by upper secondary (23%), lower tertiary (22%), and higher tertiary and above (20%). The mean financial security score increases with each level of education. In terms of living with a spouse or partner, 70% do so, while 30% do not. Among research persons cohabitating with a spouse or partner, the average financial security score is higher than the mean (0.168). Among those not cohabitating, the average financial security score is below the mean (− 0.398).

Table 7 presents the results of the regressions testing financial security and nationality’s influence on the three health outcomes. Based on the adjusted R^2^s, we see that the mental health model is the best fit, and explains 18% of the variance of Model 4, followed by physical health (13%) and social health (8%).

**Table 7:** The association between financial security and health outcomes (Models 1, 2, 3, 4)

	Mental health				Physical health				Social health			
	Model 1	Model 2	Model 3	Model 4	Model 1	Model 2	Model 3	Model 4	Model 1	Model 2	Model 3	Model 4
Financial security	0.361*** (0.0209)		0.371*** (0.0313)	0.281*** (0.0342)	0.218*** (0.0218)		0.352*** (.0328)	0.334*** (.0352)	0.169*** (0.0222)		0.176*** (0.0334)	0.185*** (.0363)
Nationality (= Spain)		−0.349*** (.0441)	−0.291*** (0.0413)	− 0.298*** (0.426)		− 0.119*** (0.0445)	− 0.150*** (0.0433)	− 0.147*** (0.0437)		− 0.266*** (0.0445)	−0.240*** (0.0441)	− 0.203*** (0.0454)
Interaction term (nationality and financial security)			−0.040 (0.0418)	0.006 (0.0425)			−0.227*** (0.0437)	−0.245*** (0.0436)			− 0.029 (0.0445)	−0.082* (.0451)
Gender (= man) ref. category: woman				0.052 (0.0412)				0.056 (0.0423)				−0.023 (0.0438)
Age												
25–35 years				ref.				ref.				ref.
36–45 years				−0.093 (0.0602)				−0.196*** (0.0618)				−0.117* (0.0640)
46–55 years				−0.225*** (0.0591)				−.383*** (0.0605)				−0.208*** (0.0627)
56–65 years				−0.032 (0.0602)				−0.576*** (.0619)				−0.137** (0.0643)
Education level												
Lower secondary and below				ref.				ref.				ref.
Upper secondary				0.001 (0.0576)				0.169*** (0.0591				0.112* (0.0613)
Lower tertiary				0.071 (0.0582)				0.136** (0.0598)				0.207*** (0.0621)
Higher tertiary or above				0.028 (0.0628)				0.199*** (0.0645)				0.304*** (0.0668)
Cohabitating with spouse/partner ref. category: not cohabitating				0.327*** (0.0484)				−0.096* (0.0496)				−0.238*** (0.0515)
Financial security in childhood				0.081*** (0.0193)				0.072*** (0.0197)				.0445** (0.0204)
Adjusted R^2^	0.1292	0.0300	0.1499	0.1792	0.0470	0.0025	0.0640	0.1301	0.0281	0.0172	0.0416	0.0755

* *p* ≤ 0.10, ** *p* ≤ 0.05, *** *p* ≤ 0.01; Robust standard errors are included in parentheses

In Model 1, financial security is a positive, significant predictor of all three health outcomes, at α = 0.01. For mental health, we find that a 1 standard deviation increase in financial security is associated with a 0.361 standard deviation increase in mental health. This is a 0.218 standard deviation increase in physical health, and a 0.169 standard deviation increase in social health. We therefore find evidence in support of our first hypothesis.

Similarly, in Model 2, nationality (being Spanish relative to being Dutch) is negatively associated with all three health outcomes, and is significant at α = 0.01. For mental health, we find that being Spanish is associated with a 0.349 standard deviation decrease in mental health. For physical health, this is a 0.119 standard deviation decrease. For social health, it is a 0.266 standard deviation decrease.

The picture becomes less clear-cut in Models 3 and 4, however. The base nationality variables remain relatively robust across all models and health outcomes. However, the effect of the base financial security score is different for each of the health outcomes in the presence of interaction terms (Model 3), and interaction terms and additional covariates (Model 4). For mental health, the financial security coefficient in Model 4 (0.281) is lower compared to Model 3 (0.371) and Model 1 (0.361). For physical health, the financial security coefficient is higher in Models 3 (0.352) and 4 (0.334) compared to unadjusted Model 1 (0.218). For social health, the financial security coefficient remains comparatively robust across Models 1, 3 and 4 (0.169, 0.176, and 0.185, respectively).

The interaction terms also behave differently across health outcomes. For mental health, we do not find a significant effect of the interaction between financial security and nationality. Moreover, for physical health and social health, we find that the interaction terms is negative, indicating that financial security’s association with these health outcomes is weaker in Spain than in the Netherlands. This is highly significant at α = 0.01 for physical health, with the relationship between financial security and physical health decreasing by 0.227 for Spanish research persons in Model 3, and by 0.245 for Spanish research persons in Model 4. For social health, these figures stand at − 0.029 (Model 3) and − 0.082 (Model 4). However, the interaction term is not significant in Model 3 and is marginally significant (at α = 0.10) in Model 4. We therefore do not find evidence for our second hypothesis that the relationship between financial security and health is stronger in Spain than in the Netherlands. 

Overall, we find strong evidence that financial security is related to mental, physical and social health. However, the effect of being Spanish or Dutch is less straightforward: while being Spanish relative to being Dutch itself is significantly associated with negative health outcomes, the relationships between financial security and physical and social health are weaker for Spanish research persons than for Dutch ones. For physical health, this is highly significant.

## Discussion

With this study, we join an emerging body of literature that assesses the extent to which a loss of financial security is associated with health outcomes in the context of the Great Recession. Our study addresses some of the shortcomings of existing research: we employ multifaceted definitions of health and financial security, and compare two countries with different experiences of the Great Recession. This paper represents a first step in understanding how the relationships between financial security and health outcomes may differ by context.

### Interpretation of findings

For the three health outcomes, we find highly significant, positive relationships between financial security and health, a finding widely supported by literature. We also find that being Spanish relative to being Dutch worsens health outcomes. There is ample evidence of countries with different welfare systems having different health outcomes. For instance, Eikemo et al. [14] found that Southern European countries (including Spain) had among the worst health inequalities in Europe, second to Eastern Europe. Southern European countries also had the highest prevalence of poor/fair self-rated health [14]. In contrast, the ‘Bismarkian’ countries, including the Netherlands, had the lowest health inequalities, although average prevalence rates of poor/fair self-rated health.

However, contrary to our expectation, being Spanish weakens the relationship between financial security and physical health and, to a lesser extent, social health. We find no moderating effect of nationality for the relationship between financial security and mental health. Again, because of the weaker social security system in Spain relative to the Netherlands, as well as the greater income inequality in Spain relative to the Netherlands, we initially expected that financial security would play a larger role in determining health outcomes in Spain than in the Netherlands. We find several possible explanations for our somewhat surprising findings.

First, we explore the possibility that we have found a true effect. Unemployment and underemployment are much more democratic in Spain than in the Netherlands. In 2014, the overall unemployment rate in Spain was 24.6%. For young people, the unemployment rate was 57.6%. In addition to these already-high unemployment figures, Spain in 2014 had an extremely precarious labor market, with a high number of temporary job contracts and depressed wages [44]. A lack of financial security is clearly more widespread in Spain than in the Netherlands. Given that many more people are financially precarious in Spain than in the Netherlands, it may be that nationality itself, rather than financial security, explains the differences in health outcomes.

Second, the ESS7 was undertaken only a few years after the Great Recession’s apex in 2011. Therefore, this survey may have not registered the effects of more pronounced financial insecurity on health. It may be that financial security has or will become a more important predictor of Spanish research persons’ health outcomes in the future, but this had not yet become evident. We see some evidence for this explanation with mental health, which stands out as the only health outcome that does not have a weaker effect in Spain than in the Netherlands. Mental health has been found to be one of the first aspects of health to change in response to unemployment, as opposed to measures of physical health such as mortality [45]. It may be that, as time passes, the relationships between financial security and health outcomes become stronger.

It is also worth emphasizing that the health outcomes we study likely have important similarities regarding their aetiologies. For instance, social health has been shown to be a protective factor against ill health. In a literature review, Kawachi & Berkman [46] demonstrated that social ties often helped to maintain psychological well-being (although there are some exceptions to this, especially among lower SES women, for whom social networks may be stressors). Social networks have also been identified as important agents in physical health behavior change [47]. This is perhaps why social health is less well-explained (based on the models' adjusted R^2^s) by financial security than mental health and physical health. Further, mental health and physical health may also be related. There is some evidence that mental health is on the causal pathway between lifestyle and behavior on the one hand, and physical health on the other [48]. Instead, it may be that these three types of health interact with and impact one another. Indeed, this interrelatedness is in line with Huber et al.’s definition of health as a dynamic process [23].

### Strengths and limitations

Our study has several unique features that add value to the debate of financial security’s association with health outcomes in the context of the Great Recession. First, we compare countries with different experiences of the Great Recession. A handful of existing studies (Faresjo et al. [29] comparing Greece and Sweden; Tapia Granados & Rodriguez [49] comparing Greece, Iceland and Finland; and Vandoros et al. [30] comparing Greece and Poland) have done so, but all are somewhat limited in their approaches. For instance, all have used Greece as an example of the Great Recession’s ill effects. Greece may be an outlier regarding the influence of financial security on health, so its experience may not be generalizable to other countries [50]. Other countries that fared among the worst in Europe, including Spain, Portugal, Ireland, Italy and Cyprus, may therefore be useful to explore in comparative perspective. Additionally, each of these three studies compared Greece to a country that fared relatively well during the Great Recession, but that is not in the Eurozone. It may be that these studies were examining countries that were too different to yield a meaningful comparison. By selecting two Eurozone countries with less extreme differences both before and after the Great Recession, we address this shortcoming. 

Second, our study also stands out for its more elaborated definitions of health and financial security. A majority of studies on the topic have used single variables, often from a single question of self-rated health [11]. Instead, we employ multi-faceted definitions of health and financial security. We found the ESS7 especially well-suited to our purposes, because it contained an extensive rotating module on health inequality.

Third, our study is unique in our use of the ESS. While many of the existing studies on the Great Recession’s influence on health make use of routine data sources, none have used the ESS. Instead, a large share exploited the European Union Statistics on Income and Living Conditions (EU-SILC) [51]. We therefore add a new perspective to the understanding of financial security on health.

Still, the choice of the ESS may have its downsides, as it is cross-sectional (the EU-SILC, by contrast, is longitudinal). Using a longitudinal dataset would have, among other things, enabled us to estimate more accurate model parameters, and to control for omitted variables. Our results should therefore be interpreted as associations, versus causal relationships.

Another limitation is that the ESS was not designed specifically to assess the association of financial security with health. This may mean that questions were asked in a certain way that did not yield accurate results, and/or that certain questions that were theoretically relevant were not asked. This is particularly evident with our measure of financial security: it is largely based on income security. This measure could have been more holistic if measures of housing security and occupational sorting were included. Regarding occupational sorting, some low-paid occupations confer higher levels of social prestige and job security, and are therefore often considered to be higher SES. For example, according to the National Readership Survey class system designed for the United Kingdom, clergy are in the top grade (likely due to the higher social standing and educational requirements), despite their comparatively low salaries [52]. Not taking into account occupational sorting may have caused us to less accurately and/or less completely measure the relationships between financial security and health.

Finally, our study offers an initial test of the associationof financial security with health in countries that have different experiences of the Great Recession. To adequately zoom in on these effects, we compare two countries. Of course, other comparisons are also possible, and are essential to more fully understand how financial security is differently associated with health across Europe. For instance, how do these relationships compare in Germany and Portugal? Our study is an important first step to understanding how the relationship between financial security and health may differ in different contexts, but does not offer a definitive answer as to how or why this is the case.

To address these shortcomings, and to more accurately identify the strength and direction of the relationship between financial security and health outcomes in the aftermath of the Great Recession, we suggest using a dataset that has a panel structure, more fully reflects the abstract concepts of financial security and health, and has been conducted over a longer time horizon. Doing so while comparing different countries could also make clearer how financial security’s influence on health may vary by context.

### Conclusion

Overall, we find compelling evidence that financial security is highly associated with health outcomes, and that health outcomes are often worse in Spain relative to the Netherlands in the aftermath of the Great Recession. We accomplish this by using the cross-sectional data from the European Social Survey 7. We employ multi-faceted definitions of wealth and health, and a cross-country comparison. These two features that are underutilized in the study of financial security’s association with health. Our results show that financial security is a relatively large and significant predictor of health outcomes. Also, being Spanish relative to being Dutch is associated with worse health outcomes. However, being Spanish relative to being Dutch weakens the relationship between financial security and health. Our study is an important initial step to understanding how the Great Recession may have varied relationships to health in different countries. Future studies may bring these differences into sharper relief.

## Data Availability

The European Social Survey is publicly available at: https://www.europeansocialsurvey.org/data/.
